# Green Enhanced Oil Recovery for Carbonate Reservoirs

**DOI:** 10.3390/polym13193269

**Published:** 2021-09-25

**Authors:** Bashirul Haq

**Affiliations:** Department of Petroleum Engineering, King Fahd University of Petroleum and Minerals, Dhahran 31261, Saudi Arabia; bhaq@kfupm.edu.sa

**Keywords:** green enhanced oil recovery (GEOR), green surfactant-polymer (SP) flood, carbonates, microbial enhanced oil recovery (MEOR)

## Abstract

Green enhanced oil recovery (GEOR) is an eco-friendly EOR technique involving the injection of specific green fluids to improve macroscopic and microscopic sweep efficiencies, boosting residual oil production. The environmentally friendly surfactant-polymer (SP) flood is successfully tested in a sandstone reservoir. However, the applicability of the SP method does not extend to carbonate reservoirs yet and requires comprehensive investigation. This work aims to explore the oil recovery competency of a green SP formulation in carbonate through experimental and modelling studies. Numerous formulations of SP with ketone, alcohol, and organic acid are selected based on phase behavior and interfacial tension (IFT) reduction capabilities to examine their potential for enhancing residual oil production from carbonate cores. A blending of nonionic green surfactant alkyl polyglucoside (APG), xanthan gum (XG) biopolymer, and butanone recovered 22% tertiary oil from the carbonate core. This formulation recovered more than double residual crude than that of the APG, XG, and acetone. Similarly, a combination of APG, XG, acrylic acid, and butanol increased significantly more oil than the APG, XG, and acrylic acid formulation. The APG, XG, and butanone mixture is efficient with regards to boosting tertiary oil recovery from the carbonate core.

## 1. Introduction

Oil recovery methods are grouped into three main categories [1]: primary, secondary, and tertiary processes. In the primary recovery, the oil is produced by the natural reservoir energy. After depletion of natural energy, the secondary stage is introduced. The secondary stage is water flooding, pressure maintenance, and gas injection. When the secondary method is no longer economically viable, the tertiary oil recovery, commonly known as the enhanced oil recovery (EOR), is implemented. According to Lake et al. (2014) [2], the EOR methods are chemical, gas, thermal, etc. Chemical EOR [1] processes involve injecting specific liquid chemicals that effectively displace oil because of their phase-behavior properties, decreasing the interfacial tension (IFT) between the displacing liquid and oil. The popular chemicals are surfactants, polymers, and alkaline. The primary displacing liquid slug is a complex chemical system called a micellar solution. The slug contains a surfactant, co-surfactant (alcohol), oil, electrolytes (i.e., NaCl), and water. Surfactant slug is followed by a mobility buffer called polymer solution. The primary mechanism of the chemical EOR is a reduction of IFT between oil and water/brine. The gas injection EOR method displaces crude with a miscible fluid and forms a single phase when mixed at all proportions with the oil at the existing condition. The process uses gases, for instance, carbon dioxide (CO_2_), nitrogen (N_2_), and natural gas, mainly methane (CH_4_) and flue gas. The injected gas expands in the reservoir and pushes additional oil to the production well or the gas dissolves in the oil to lower the viscosity and IFT and improves the recovery. Thermal methods include injecting hot water, steam, and other gas by conducting combustion in situ of the oil. The procedures apply heat to increase the temperature of the crude oil in the formation and reduce oil viscosity and/or vaporize part of the oil, thereby decreasing the mobility ratio and increasing oil recovery. Thermal EOR methods are generally applicable to heavy and dense crudes. Other processes [1] include microbial enhanced oil recovery (MEOR). MEOR [3] is an environmentally friendly enhanced oil recovery method that involves injecting microorganisms and produces surfactant, polymer, alcohol, ketone, acids, and gas in situ and enhances oil recovery. According to Haq [3,4], the green enhanced oil recovery (GEOR) is an eco-friendly EOR technique that involves the injection of specific green fluids, for example, surfactants, polymers, alcohols, acids, ketones, and gas (N_2_, CO_2_) that effectively improves macroscopic and microscopic sweep efficiencies as a result increases residual oil recovery. Classification of GEOR, surfactant, and polymer flooding mechanisms and challenges in carbonates, a summary of literature review, knowledge gaps, the objective of the work, and an outline of the research are discussed in the following sections.

**Figure 1:**
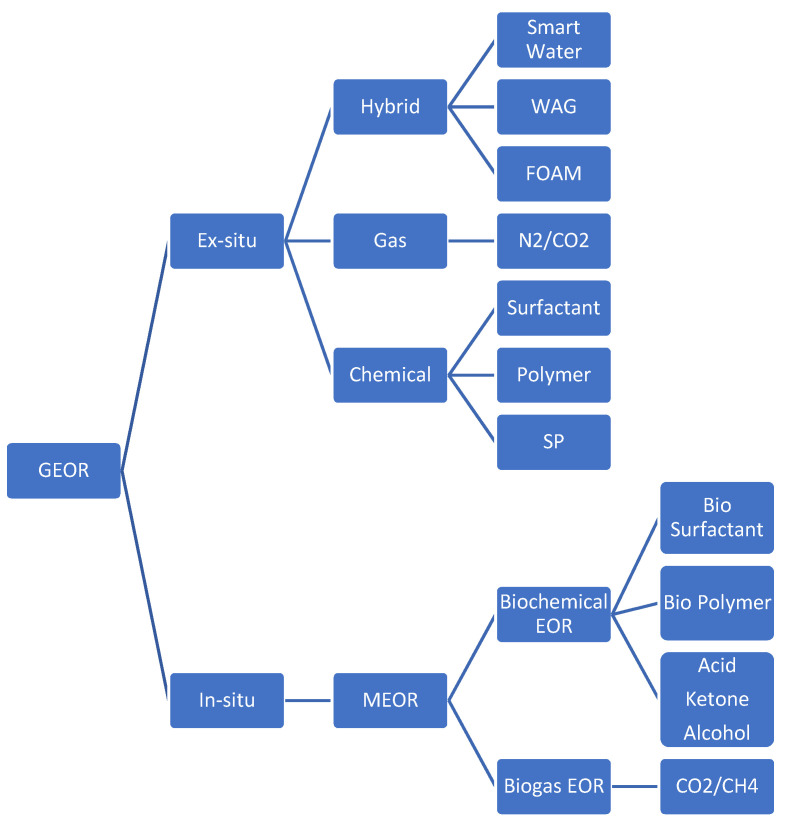
GEOR classification.

GEOR processes [3] can be grouped into two categories: in situ and ex situ. Regarding in situ methods, bacteria with nutrients are injected into a reservoir, producing by-products such as surfactants, polymers, alcohols, ketones, acids, and gases (i.e., CO_2_ and CH_4_). These products increase microscopic and macroscopic displacement efficiencies and boost oil production. Microbial enhanced oil recovery (MEOR) is one example of an in situ GEOR method. Ex situ GEOR processes involve the injection of green gases and/or chemicals that improve microscopic and macroscopic displacement proficiencies and enhance oil recovery. Ex situ methods can be subdivided into three main categories: chemical, gas, and hybrid. Smart water, water alternating gas (WAG), foam, and surfactant-polymer (SP) flooding are all hybrid in nature. N_2_ and CO_2_ floods are grouped as gas flooding. Nature-friendly chemicals are used in green chemical enhanced oil recovery (GCEOR). GCEOR involves injecting specific eco-friendly chemicals such as surfactants, polymers, alcohols, ketones, and acids. In the GCEOR process, the primary displacing liquid slug is a complex chemical system called a micelle solution that contains nature-friendly surfactants, co-surfactants, oil, electrolytes, and water. The GEOR process is shown in Figure 1.

The surfactant flooding technique involves injecting surfactants to mobilize oil via IFT reduction and wettability alteration [5]. The surface-active agent, commonly known as surfactant, can change the rock’s wettability and decrease the IFT between oil and micellar solution [6]. This mechanism led to producing more oil from the formation. The governing mechanism of surfactant flooding is achieved by forming the microemulsion phase between the oil and water phases. The emulsion formed by surfactant at the fluid interface is dependent on the emulsion rheological properties, phase behavior, salinity, initial volume ratio, and internal structure. The primary mechanism of polymer flood is to improve the aqueous phase viscosity (μ_w_) and the effective water permeability (k_w_) [7]. Mobility ratio (M) is calculated as

M=λwλo=kwμwkoμo

where $\lambda_{w}$ = brine mobility, $\lambda_{o}$ = oil mobility, $k_{w}$ = effective permeability of brine, $k_{o}$ = effective permeability of oil, $\mu_{w}$ = brine viscosity, and $\mu_{o}$ = viscosity.

The mobility ratio determines the flooding performance of the polymer. Low values of mobility ratio (M < 1) according to Lai (2007); Sorbie and Phil (1991) [8,9] indicate a piston-like displacement and good sweep efficiency while (M > 1) leads to more improvement in the sweep efficiency by the fluids.

There are many challenges of surfactant and polymer flooding in carbonates. The carbonate reservoirs are composed of dolomite, calcite, magnesite, anhydrite, and gypsum rocks. The carbonate formations typically show low porosity, and the mixed wet rock characteristics and fractured composition that leads to low oil recovery [10]. One of the main challenges of the surfactant flooding method is adsorption, which causes economic feasibility [10]. Many studies on a range of carbonate reservoirs with different mineralogy have been conducted to quantify adsorption phenomena and suitability for EOR [9,11,12,13,14]. The other challenges of surfactant flooding in carbonates are precipitation, gravity segregation, and phase trapping. The factors that affect the adsorption process are temperature, rock mineralogy, fluid saturations, salinity, and divalent cations. The surfactant adsorption is controlled by the structure, which must be accounted for in the design phase. Phase trapping involves the migration of the surfactant to either the oil or microemulsion phase. High salinity and temperature may cause the surfactant to transfer to the oil face, thus reducing the effectiveness of the flooding process and surfactant loss. On the other hand, the challenges of polymer flooding encountered in carbonate reservoirs are degradation, high temperature, salinity, and complexity. Degradation is a process that would break down the molecular structure of the macromolecules in the polymer solution resulting in a partial or complete loss of viscosity. Note that the polymers are expected to be stable throughout their propagation during polymer flooding. The carbonate reservoirs with high well spacing experience the major degradation challenge regardless of the kind of degradation, for example, chemical, thermal, mechanical, or biological [15]. It has been suggested that polymer flooding has the potential to increase oil recovery from the carbonates [16,17,18]. However, it becomes challenging to find suitable polymers for carbonates due to the complexity of the reservoir and high salinity and temperature. In addition, degradation of polymer is more in elevated temperature and salinity. According to Roehi and Choquette (1985) [19], the world’s known oil reserves in carbonate reservoirs is ~50%. The majority of the carbonate reservoirs are naturally fractured, poorly understood, and have lower recovery than an unfractured reservoir. Oil recovery in carbonates mainly depends on matrix permeability, wettability, fracture intensity, and fluid properties. The vital recovery controlling parameter is the wettability of the carbonate reservoir [20,21].

The challenges of previous studies in surfactant based chemical EOR can be summarized as follows: Most of the publications related to surfactant flooding found in literature deal with synthetic surfactants. These floodings are applied mainly in sandstone reservoirs. A relatively smaller number of surfactants flooding are found in the literature in carbonate, and these surfactants are synthetic. A significant portion of these surfactants is not environmentally friendly. A similar trend is observed in polymer flooding. Most of the polymers applied in chemical EOR are either HPAM or PAM, and they are synthetic. There are few publications on xanthan gum biopolymer, and the polymer is also used in a sandstone reservoir. The application of biopolymer in carbonates is restricted due to harsh reservoir conditions. Several publications of SP or ASP flooding in carbonate in tables deals with chemicals such as surfactant, polymer, alcohol, and alkali. These are synthetic, and many of them are not eco-friendly. There is a knowledge gap in environmentally friendly SP formulation. In addition, there is not enough information available on the combined effect of a green surfactant, alcohol, ketone, acid, and polymer on EOR in carbonates. It would be better to have a green SP formulation for carbonate reservoirs for sustainable development of the petroleum industry and requires step-by-step documentation to understand the role of green SP in carbonate.

The project aims to (a) determine optimum salinity through phase behavior modelling and (b) examine the combined impact of green surfactant, polymer, organic acids, and microbial products on reducing interfacial facial tension and boosting residual oil recovery from a carbonate core. The goals are achieved by meeting several research milestones, which are the results of several experiments and modeling. These are IFT measurement, phase behavior study, core flooding experiment, and mathematical modeling.

## 2. Literature Review

### 2.1. Surfactant Flooding in Carbonates

Surfactant flooding is an enhanced oil recovery (EOR) technique involving surfactant injection to mobilize oil via IFT reduction and wettability alteration. The main challenge in surfactant flooding is adsorption that leads to economic viability issues. The review focuses on the prime challenges.

The surfactant’s adsorption is caused by the electrostatic interaction affecting the mineralogy of the rock and structure of the surfactant [22]. Mannhardt et al. (1994) [23] found that adsorption could be minimized by adding cationic surfactant with low affinity with low to moderate brine. An experimental study was conducted by Trogus et al. (1977) [24], and it was found that the adsorption rises sharply with increasing nonionic surfactant concentration. It raised to a maximum point corresponding to the critical micelle concentration (CMC) and remained constant. It is also noted that the reduction of adsorption could be achieved by adding alcohol in the surfactant solution. They also conducted a modelling study of the adsorption process and found a minor dispersion effect in anionic surfactants. The adsorption was more in kaolin than sandstone. It was believed that the adsorption of cationic surfactant is less in carbonate core than sandstone [14]. Tabatabal et al. (1993) [25] found that the adsorption of cationic surfactant on carbonate core was relatively low because the mineral surface had a lattice charge.

Ahmadi and Sheng (2016) [11] carried out a surfactant flooding operation on carbonate reservoir samples to quantify the applicability of nano-surfactant extract from *Ziziphus Spina-Christi* leaves. Findings revealed that an increase in surfactant concentration and hydrophilic nano-silica concentration in the surfactant solution increases the ultimate recovery of the process.

Researchers found a number of ways to minimize adsorption: (1) low concentration surfactant injection, (2) pre-flush with a sacrificial chemical, and (3) co-injection of surfactant and co-surfactant. Polyacrylate works better as a sacrificial agent for carbonate and sandstone rocks and is independent of surfactant types. It is found that adsorption decreases with the increasing molecular, for example, sodium-based surfactants [26]. Krumrine et al. (1982) [27] performed an experimental study and found that adsorption could be reduced by adding sodium silicate, sodium carbonate, and sodium tripolyphosphate. Three minerals—dolomite, calcite, and kaoline—were selected to measure adsorption using CO_2_ injection at low and high pressure [12]. The results revealed that the tertiary amine Ethomeen C12 (anionic surfactant) converts to cation at high pressure (pH = 4) and reduces adsorption. At high pressure, divalent and trivalent cations decrease the dissolution of calcite and dolomite, leading to an increase in adsorption [13]. Tsau et al. (2000) [28] described that lignosulfonate as pre-flush gives more economic advantage when used as pre-flush than co-injection to reduce adsorption.

In summary, these challenges can be resolved by one of these techniques: (a) The salinity gradient technique involves injecting low salinity brine to change the high salinity state to the optimum salinity required for the successful implementation of surfactant flooding, followed by injection of surfactant slug [29]. (b) Use of sacrificial agents. The sacrificial agents have been proposed by many researchers to reduce adsorption and improve the flooding process’s effectiveness. Agents such as polystyrene sulfonate, sodium polyacrylate, calcium lignosulfonate, and polyacrylate bind to the active adsorption sites available prior to the surfactant injection. (c) Alkali-surfactant flooding. In this process, alkali is added as an adsorption reducing agent. Additionally, the alkali also generates in-situ surfactants from naphthenic acids in crude oil. (d) Surfactant foams. The method involves a surfactant aided CO_2_ flooding process. However, this has concerns about stability in the reservoir. Surfactant flooding is commonly seen in a sandstone reservoir. A list of surfactants applied in carbonate and sandstone is presented in Table A1 in Appendix A. It indicates that most of the fields use a synthetic surfactant. Two fields out of thirteen (~15%) use biosurfactants, and four are carbonate formation.

### 2.2. Polymer Flooding in Carbonates

Polymer flooding is a popular EOR method in oil industry [7] and is suitable for sandstone reservoirs. However, there are many challenges faced in carbonate reservoir due to high temperature and salinity, and low permeability. In recent years, researchers have been working toward extending this success to carbonate reservoirs in more challenging reservoir conditions in terms of temperature, salinity, and permeability. The review discusses some significant challenges: polymer degradation, salinity, and temperature.

Polymer degradation is a process that would break down the molecular structure of the macromolecules in the polymer solution resulting in a partial or complete loss of viscosity [7]. Note that the polymers are expected to be stable throughout their propagation irrespective of the type of degradation (chemical, thermal, mechanical or biological). Sandengen et al. (2017) [15] describe that carbonate reservoirs with high well spacing experience this major challenge. They cited an example of the giant carbonate offshore fields where the residence time inside the reservoir could be in years.

Traditionally, it is essential to mention that polymer flooding is sensitive to elevated temperature and salinity [30]. However, pre-flush of low salinity make brine may lead to favorable condition polymer flood which could reduce chemical degradation of polymers. Alfazazi et al. (2019) [30] described elaborately that HPAM might be applied to pre-flushed HTHS carbonate reservoir to improve oil recovery. A study by Diab and Al-Shalabi (2020) [7] shows that two conditions temperature and salinity. These are usually encountered in carbonate reservoirs worldwide, particularly in the Middle East, to apply polymer flooding.

A study on screening workflow for carbonate green field by Al-Shalabi et al. (2018) [31] mentioned how their screening ranking was performed based on pore-scale, compatibility of polymer with rock surface, macro-scale filtering, and industry guidance. Diab and Al-Shalabi (2020) [7] investigated the importance of the screening stage in carbonate reservoirs. They stated how vital this stage is in carbonate polymer flooding for EOR purposes. Screening tests, according to them, are inexpensive and provide enough knowledge about the solution rheology, thermal stability, and salinity tolerance. Some of the most important screening tests are viscosifying ability, long-term thermal stability, sensitivity for salinity and hardness, shear stability, and static adsorption. The field application of polymer is listed in Table A2, and it reveals that the field application of biopolymer is ~19% (3 out of 16), and all fields are sandstone reservoirs.

### 2.3. SP Flooding in Carbonates

SP is commonly known as surfactant-polymer. The SP flooding method involves injecting surfactant and polymer (SP) to alter EOR properties and increase residual oil production. The main issue in SP flooding is chemical losses due to adsorption, surfactant precipitation, and cost. These issues can be reduced by the injection of alkali, which is a cheap chemical. When SP blends are injected with alkali, the technique is referred alkaline surfactant polymer (ASP) flooding. The SP or ASP flooding technique applied in the carbonate fields is listed in Table A3. Experimental studies are summarized in Table A4. It shows that all SP and ASP formulations are synthetic. It would be better to have an eco-friendly formulation for carbonates.

In 2020, few publications on ASP gave exciting information, and these are discussed here. Ahmed and Shenj [32] conducted experiments and simulation studies of foam assisted ASP flooding. They examined the oil recovery performance of different injectivity scenarios of foaming agents and ASP formulation. The results revealed that ASP produced maximum incremental oil due to the gas phase. Fei Pan et al. [33] studied cationic and anionic surfactants’ role on IFT and oil recovery. They found that the anionic linear alkylbenzene sulfonic acid (LABSA) surfactant produced 15% more oil than the cationic Cetyl trimethyl ammonium bromide (CTAB) surfactant. LABSA has a higher IFT effect than CTAB. Afshin Davarpanah [34] conducted a parametric study of nanoparticles assisted polymer flooding in evaluating injectivity performance of two-phase flow. It was found that the higher mobility ratio and polymer concentration gave higher oil recovery.

The primary limitation of the earlier work on chemical EOR can be summarized as follows:

1. The majority of the publications of surfactant flooding in carbonates deals with synthetic chemical surfactants and co-surfactants. Many of them are not eco-friendly, either surfactant or co-surfactant. Corrosive acid is usually used to increase the productivity of the formulation. Several publications deal with environmentally friendly surfactants and co-surfactants in oil recovery. However, these formulations are applied in a sandstone reservoir. There is not enough information available on the combined effect of a green surfactant, alcohol, ketone, acid, and polymer on EOR in carbonates.
2. Polymer flooding, especially synthetic polymer, is a widely used chemical oil recovery method in sandstone reservoirs. Most of the polymers applied in chemical EOR are synthetic such as HPAM or PAM. There are few biopolymers, such as Xanthan gum, used in the sandstone reservoirs. The application of biopolymer in carbonates is restricted due to harsh reservoir conditions.
3. Surfactant-based polymer flooding is widespread in sandstone reservoirs but limited in carbonate reservoirs. Several publications of SP or ASP flooding in carbonate in tables deals with chemicals such as surfactant, polymer, alcohol, and alkali. These are synthetic, and many of them are not eco-friendly. There is a knowledge gap in environmentally friendly SP formulation. It requires a step-by-step documentation to understand the role of green SP in carbonate.

## 3. Carbonate Reservoirs in Saudi Arabia

The Kingdom of Saudi Arabia (KSA) is one of the largest countries in Asia. Since the first oil discovery in March 1938 at the Dammam oil well No. 7 at a depth of 1440 m, Saudi Arabia has become a major supplier of crude oil worldwide. The Saudi Arabian hydrocarbon accounts for ~50% of the GDP as well as 85% of export earnings [35]. Saudi Arabian oil reserves are the second-largest globally and are estimated to be at 268 billion barrels [36]. Some of the major oil fields in Saudi Arabia are as follows.

### 3.1. Abqaiq

Abqaiq is the northeast extension of the Ghawar field, the largest oil field globally. The main oil-producing section in the Abqaiq field is Arab-D, mainly composed of limestone and dolomitic mixture. The depth of Arab-D formation is 4400–4700 ft, and the temperatures in these reservoirs can be found to be around 196 °F. The current reservoir pressure in this formation has reached 2143 psi. The Arab-D section of the Abqaiq field has a relatively high permeability of about 80–150 md, and the porosities range from 5 to 30%. The crude is light crude with an API of about 30.5. The salinity of the reservoir fluid ranges between 38,000 and 240,000 ppm. There are currently 107 wells in operation, and the recovery mode is waterflooding [37,38,39]. Below the Arab-D formation in the Abqaiq field is the Hanifa formation, which can be found at a depth of 5200–7500 ft. The Arab-D reservoir separates it by about 450 ft of impermeable carbonates of the Jubaila formation. An engineering estimate can be made regarding the temperature of the formation to be around 237 °F. The porosity of the formation is similar to Arab-D and is approximately 5–30%. It has a very low permeability of approximately 0.1–10 md. The crude is Light oil with an API of ~30.5. The reservoir is mainly composed of organic-rich carbonate rocks and is recovered by waterflooding [40].

### 3.2. Haradh

Haradh is located in the eastern province of KSA and is nearly 80 km from the Arabian Gulf. It is situated in the southern part of the Ghawar field. The Haradh field is located at a 5200–7500 ft depth and is mainly composed of Carbonate rocks such as limestones and dolomites. One of the significant features of the Haradh field is the tilted oil-water contact which gets shallower from north to south. The temperature in the reservoir reaches 170 to 210 degrees Fahrenheit. The reservoir is maintained at a pressure of 2200 psi, and the bubble point pressure is about 1650 psi. Data for the permeability and porosity of the reservoir are not present, but it is known to have super permeability due to fractures. The crude oil is a light oil with an API of 33, and the salinity of the reservoir is around 37,000 to 150,000 ppm. There are 28 injections, 32 production, and 13 observation wells. Improved oil recovery (IOR) techniques include applying Intelligent wells and Maximum Reservoir Contact (MRC) wells. The field is using waterflooding techniques [40,41,42].

### 3.3. Khurais

The Khurais complex was discovered in 1970 and had the capacity to produce ~1.2 million barrels of crude oil per day. The complex consists of three fields: Khurais, Abu Jifan, and Mazalij. They are located approximately 100 miles east of Riyadh and consist of both Arab-D and Hanifa formations. Arab-D reservoir consists of clastic limestones and is a few hundred feet thick, and the Hanifa is located below it. The rock quality is poor, and the degree of fracturing is high. The pressure of the Khurais field is about 1800 psi, and the API is around 33–36. There are 50 wells in operation. Not a lot of other data for the Khurais field is present. Still, as it has both Arab-D and Hanifa formations, its properties would be similar to the Abqaiq field mentioned earlier in the report [40,41,42,43,44,45].

### 3.4. Khursaniyah

Khursaniyah is one of the oil fields situated on the coastal plain of Saudi Arabia and is a part of the Ghawar field. Khursaniyah was discovered in 1956, and regular production began when the Arab-C came online in 1960. The proven reserves of Khursaniyah are more than 1.5 billion barrels and can extend up to a depth of 7000 ft. Khursaniyah is mainly composed of carbonate rocks with a porosity of ~23% and permeabilities ranging between 80 and 250 md. Light crude oil is produced from this reservoir with an API of 31. The reservoir is currently being waterflooded to maintain pressure. Khursaniyah has also successfully implemented intelligent, latest systems and applications [46,47].

### 3.5. Qatif

In 1945, the Qatif field was discovered and started producing oil in 1946. It is situated on the western side of the Arabian Gulf. The field has seven oil-bearing reservoirs. These are Arab A to D, Hainfa, Upper Fadhili, and Lower Fadhili. Arab C and D are superior to others. Arab C produces medium crude, and D has light crude oils. The Qatif field is carbonate members of cycles that deposited marine carbonate sediments capped by regressive sabkhah anhydrite. The porosity of this field is around 12–35%, and the permeability ranges between 80 and 500 md. The produced fluid API varies between 28 and 42. The Qatif field is the first i-field in Saudi Aramco. The field is well equipped with asset measurement, communication, control, and field rate optimization [48,49].

### 3.6. Manifa

Manifa is the world’s fifth-largest oil field and is the world’s largest offshore crude oil production increment built in a single phase. The field was discovered in 1957, 200 km northwest of Dhahran, mainly offshore, with a Gulf water depth ranging from 6 to 36 feet. It is a carbonate reservoir and produces heavy oil in the range of 24 to 26 API. Not much data for the field is public, but the field’s depth is greater than 17,000 ft, and the longest well in Saudi Arabia, which is about 37,042 ft, has been drilled in this field. Extended reach wells are pretty common in this field [50].

## 4. Methodology

### 4.1. Materials

The Alkyl Polyglucoside (APG) 264 is a nonionic surfactant; the commercial name is the BASF supplied Glucopon 600 CSUP. The Xanthan gum, formic acid, acetic acid, acrylic acid, acetone, butanone, and alcohol were purchased from Sigma Aldrich (Dammam, Saudi Arabia). The physicochemical properties of acetone and butanone are given in Table 1. The acids and ketones were 99.98% pure. The NaCl was 99.87% pure and purchased from Sigma Aldrich. The Arabian light crude oil was used throughout this research and supplied by Saudi Aramco (Dhahran, Saudi Arabia). The crude is a light oil and black liquid. The density and viscosity of this crude oil were measured at 25 °C and 52 °C in our laboratory. The densities are 0.8699 g/cm^3^ and 0.8484 g/cm^3^ at 25 °C and 52 °C, respectively. Similarly, the viscosities are 19.8 cP and 8.2 cP at 25 °C and 52 °C, respectively.

**Figure 2:**
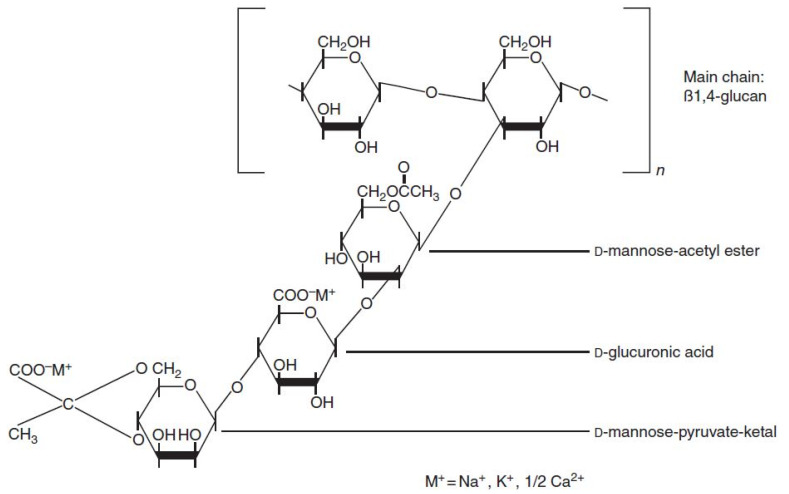
Chemical structure of xanthan gum [51].

**Figure 3:**
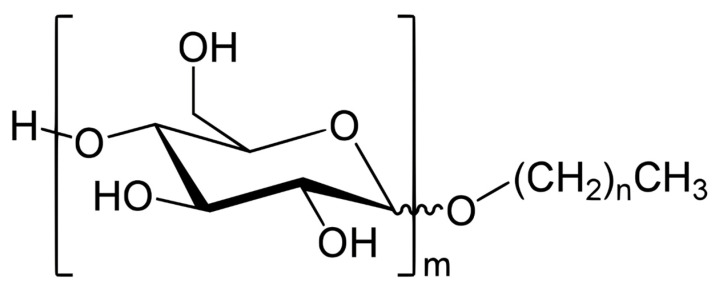
Chemical structure of alkyl polyglucoside [52].

Xanthan gum is a polysaccharide anionic polymer produced by fermentation. It is composed of a β-(1→4)-d-glucopyranose glucan backbone with side chains of (1→3)-α-d-mannopyranose-(2→1)-β-d-glucuronic acid-(4→1)-β-d-mannopyranose on alternating residues [51]. The chemical structure of XG is shown in Figure 2. APG is biodegradable and derived from sugar, glucose, and fatty alcohol. The raw materials of APG are starch and fat. The hydrophilic part of APG is typically sugar, and a hydrophobic end is an alkyl group with variable lengths [52]. The chemical structure is displayed in Figure 3 [52].

**Table 1:** Physicochemical properties of acetone and butanone [53,54,55,56,57].

Properties	Formic Acid	Acetic Acid	Acrylic Acid	Acetone	Butanone
Chemical Formula	H-COOH	CH_3_-COOH	CH_2_=CH-COOH	CH_3_-CO-CH_3_	CH_3_-CO-CH_2_CH_3_
Molar mass	46.025 g/mol	60.052 g/mol	72.06 g/mol	58.08 g/mol	72.117 g/mol
Appearance	Colorless liquid	Colorless liquid	Colorless liquid	Colorless liquid	Colorless liquid
Density	1.22 g/cm^3^	1.049 g/cm^3^	1.05 g/cm^3^	0.78 g/cm^3^ at 25 °C	0.80 g/cm^3^ at 25 °C
Boiling point	100.8 °C	118 °C	141 °C	56.05 °C	79.64 °C
Viscosity	1.57 cP at 20 °C	1.22 cP at 20 °C	1.3 cP at 20 °C	0.29 cP at 25 °C	0.43 cP at 25 °C

### 4.2. Selection of Chemical

In the microbial EOR process, tertiary oil can be recovered by reducing interfacial tension and the capillary trapping forces between oil and water with microbial products such as surfactants, polymers, alcohol, acid, and ketones. The effects of ketones and acids in green surfactant-polymer (SP) formulations in carbonates are not well understood yet require further analysis. For this reason, acetone, butanone, formic acid, acetic acids, and butanone are selected.

Alkyl polyglucoside (APG) 264 is chosen from the nonionic group because APG 264 is an eco-friendly synthetic surfactant made from coconut, palm oil, corn, potato, or wheat residues. It is entirely biodegradable. The toxicity of APG 264 is very low [52,58]. Xanthan gum (XG) is a polysaccharide, non-toxic, and biodegradable polymer. The XG is by a *Xanthomonas campestris* bacterium produced in a fermentation process. It is environmentally friendly and relatively cheaper than Schizophyllan and Sclerogucan [59]. That is why it is selected for this work.

### 4.3. Phase Behavior Modeling

The phase behavior study is an essential part for EOR application. It is very user-friendly method to predict surfactant performance. The main goals of most phase behavior studies are to identify regions of middle phase microemulsion and determine optimum salinity. The middle phase emulsion can be designed to have low Interfacial Tension (IFT) used in oil recovery processes.

The experiment was conducted in the laboratory using the concept of salinity scans and followed several steps. In this experiment, surfactant centration was fixed and NaCl concentration was varied from 1 to 7%. Each sample consists of an aqueous phase and the oil phase. It was determined that the sample size would be 9 mL, given the size of the vials: with water oil ratio (WOR) = 1, there would be 4.5 mL aqueous phases and 4.5 oil phases. The aqueous phase consisted of 3.5 mL brine and 1.0 mL surfactant. The concentration of APG 264 was 0.50%, and the NaCl concentration varied from 0 to 7%. The phase volumes were measured when they were stable and reached in equilibrium. The observed and measured phase volumes are presented in Table 2.

**Figure 4:**
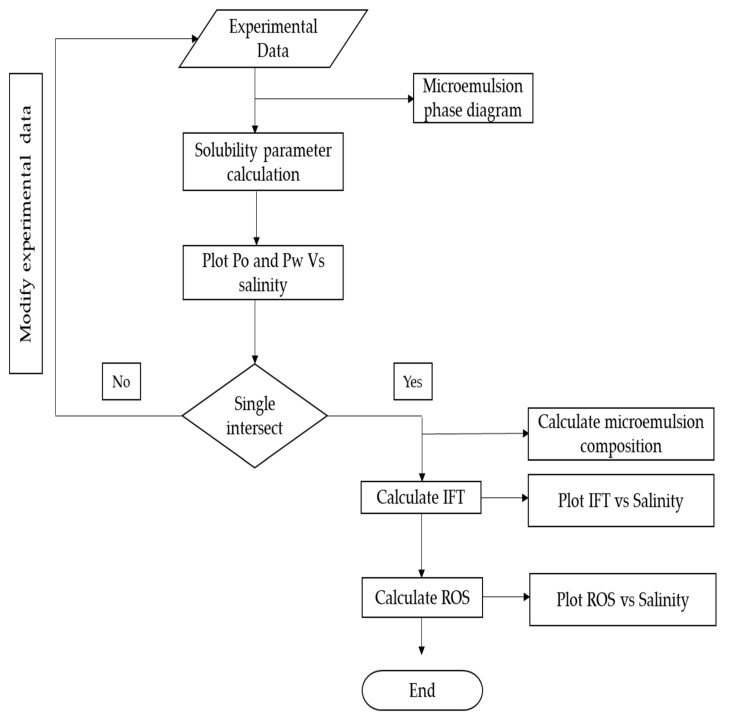
Model workflow diagram.

The development of a mathematical model for optimum salinity, interfacial tension, and residual oil calculations using experimental data and Healy and Reed correlation is shown in a process flow diagram. The workflow of the modelling is described in Figure 4. In the workflow, the solubilization parameters of oil and water in the micro-emulsion phases are first calculated using Equations (1) and (2). Then, the IFTs of oil and water in the microemulsion phases are calculated using Equations (3) and (4). After that, the solubilization parameters are plotted against salinity to obtain the optimum salinity. The optimum salinity is determined by the point of intersection between the oil and water solubilization parameter curves. Similarly, the IFTs of oil and water are plotted against salinity, and the intersecting point gives the IFT of the formulation.

(1) $$P_{o}=\frac{V_{o}}{V_{s}}=\frac{\mathrm{Volume}\;\mathrm{of}\;\mathrm{oil}\;\mathrm{in}\;\mathrm{micro}-\mathrm{emulsion}\;\mathrm{phase}}{\mathrm{Volume}\;\mathrm{of}\;\mathrm{surfactant}\;\mathrm{in}\;\mathrm{micro}-\mathrm{emusion}\;\mathrm{phase}\;}$$

(2) $${\;P}_{w}=\frac{V_{w}}{V_{s}}=\frac{\mathrm{Volume}\;\mathrm{of}\;\mathrm{brine}\;\mathrm{in}\;\mathrm{micro}-\mathrm{emulsion}\;\mathrm{phase}}{\mathrm{Volume}\;\mathrm{of}\;\mathrm{surfactant}\;\mathrm{in}\;\mathrm{micro}-\mathrm{emusion}\;\mathrm{phase}\;}$$

(3) $$\log_{\sigma_{\mathrm{mo}}}=-7.058+\frac{6.285}{0.04477\;\left(P_{o}\right)+1}$$

(4) $$\log_{\sigma_{\mathrm{mw}}}=-12.856+\frac{12.167}{0.01280\;\left(P_{w}\right)+1}$$

where

P_o_ = Solubilization parameter of oil in the micro-emulsion;

P_w_ = Solubilization parameter f water in the micro-emulsion;

$\sigma_{\mathrm{mo}}$ = IFT of oil in the micro-emulsion;

$\sigma_{\mathrm{mw}}$ = IFT of water in the micro-emulsion.

### 4.4. IFT Measurement

**Figure 5:**
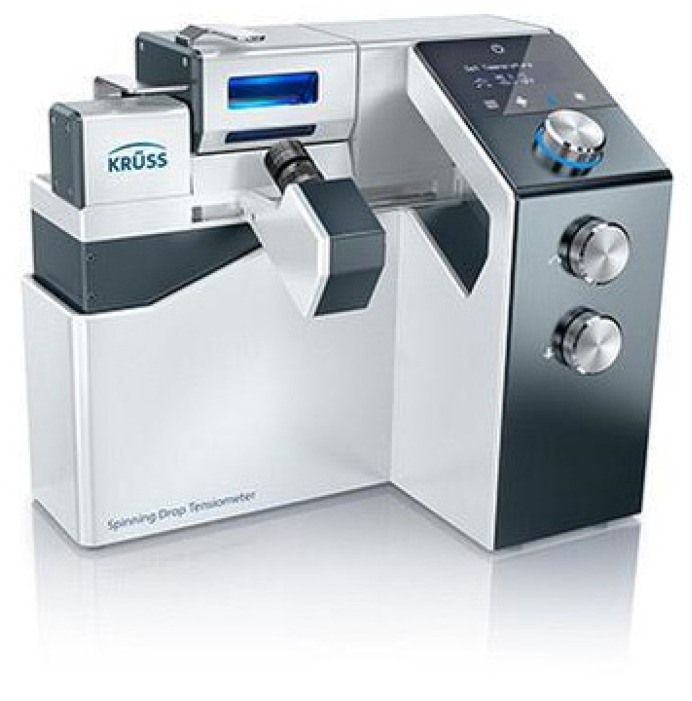
Spinning drop tensiometer (STD 100).

The Spinning Drop Tensiometer (STD) 100 from KRUSS shown in Figure 5 was used for the IFT measurements. The IFTs of the APG 264, acid, and ketone mixtures were determined to identify the optimum concentration for the core flood experiments. The density of each sample was measured at 25 °C to determine the IFT value at 25 °C. First, the cell is cleaned using hot water and soap. A 20 mL portion of each sample is required to complete a measurement. After loading the sample, the tool is calibrated before injecting the oil drop for each sample. Next, an oil drop is injected into the cell while it rotates at 500 RPM. Then, the rotation speed is set to 2800 RPM. The tool must be adjusted to centralize the oil drop. Then, adjustments are made to the temperature and camera on the tube. Then, the drop of oil is released. Finally, the IFT values are measured for at least two hours to obtain a constant IFT value. Seven samples of formic acid solution with various concentrations were prepared for IFT measurement and given in Table 7. The IFT of the 0.5% APG and 2% NaCl sample was measured when the value was closed to steady. It became nearly constant after two hours. Using this benchmark, samples 3 to 7 were measured and listed in Table 7.

### 4.5. Core Flood Experiment

Typically, a core flood experiment is performed in four phases. These are core preparation, water flood, chemical, and post floods. Five carbonate and two sandstone cores were cut and then dried for around four hours at 60 °C in an oven. After that, the dry weights of the cores were measured, and overburden pressure was applied to the core holder. Next, the plugs were placed in a vacuum chamber to remove the air. In the brine saturation process, 2% NaCl solution was applied to saturate each core, then flooded with Arabian light crude oil. Next, the oil-saturated plugs were put in aging for three days. Measured core properties such as length, diameter, pore-volume, dry weight, porosity, and permeability are shown in Table 3. After aging, the cores were ready for flooding.

**Figure 6:**
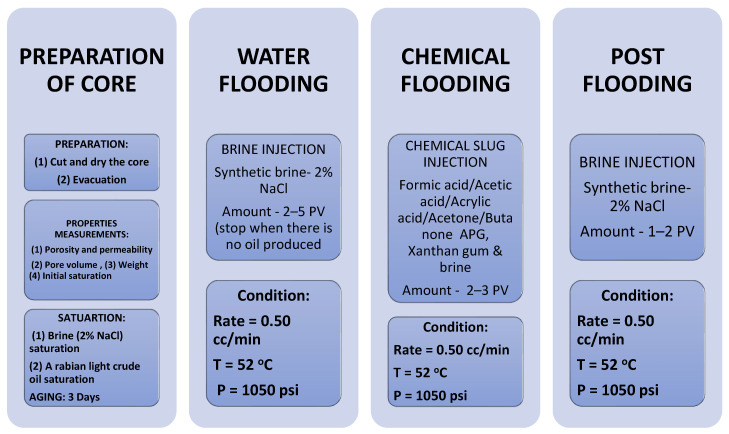
Core flooding procedure.

**Figure 7:**
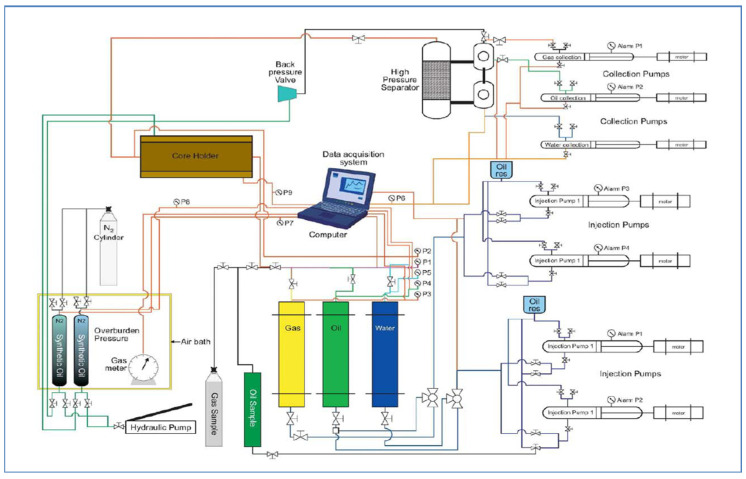
A schematic diagram of a core flood system.

First, a core plug was flooded with 2% NaCl solution about 2 to 4 PV when no oil was produced. Then, it was flooded by a chemical slug of about 2 to 3 PV. In this phase, seven formulations were flooded in seven core plugs. After that, the process was followed by a 1–2 PV post-flood with 2% NaCl solution to make sure all released oil inside the core and tube were accumulated in the measuring cylinder. Formulations are given in Tables 6–10. The flooding process is given in Figure 6. The schematic diagram of the core flood system is shown Figure 7. The equipment consists of four injection and three collection pumps and a temperature control chamber to simulate the required reservoir temperature. The core holder, separator, and injection and collection vessels are kept in the closed chamber to maintain the same temperature. There are three injection cylinders connected to the core holder through several valves to control the fluid flooding. These cylinders are crude oil, water, and gas. A manual hydraulic pump applies overburden pressure on the core to ensure no fluid passing outside the core. The pump is connected to two synthetic oil vessels to stabilize the pressure before the core inlet.

**Figure A1:**
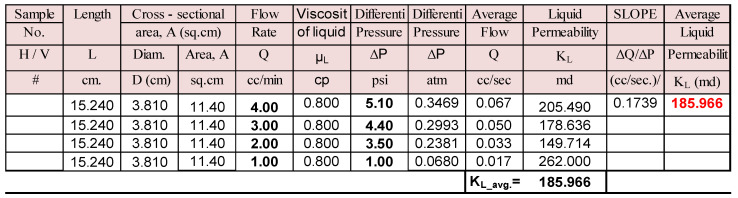
Permeability calculations.

The detailed porosity, permeability, and oil saturation calculation and data are given in Table A5 and Table A6 and Figure A1 in Appendix B.

## 5. Results and Discussion

### 5.1. Optimum Salinity Determination

Samples were prepared with 4.5 mL of Alkyl Polygluside and NaCl formulations and 4.5 mL of crude oil. All samples were kept for three weeks to reach equilibrium at room temperature and pressure after mixing well. The aqueous, middle, and oleic phases of each system were measured and recorded.

**Figure 8:**
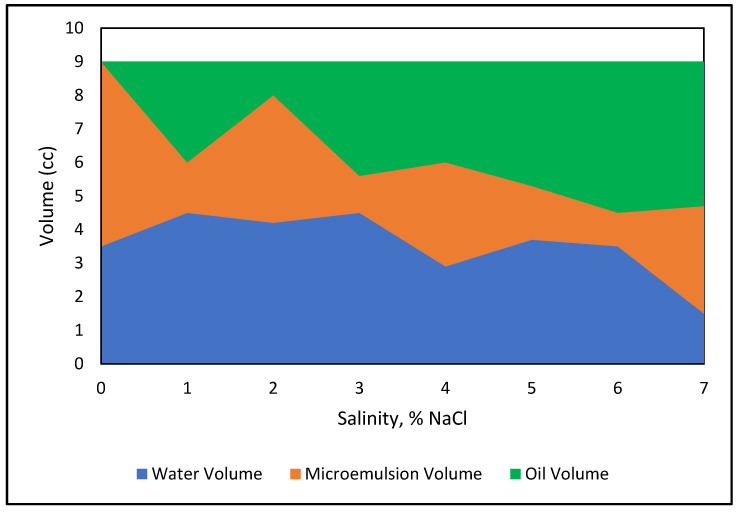
Phase diagram of 0.5% APG, crude oil, and 0–7% NaCl mixture.

**Figure 9:**
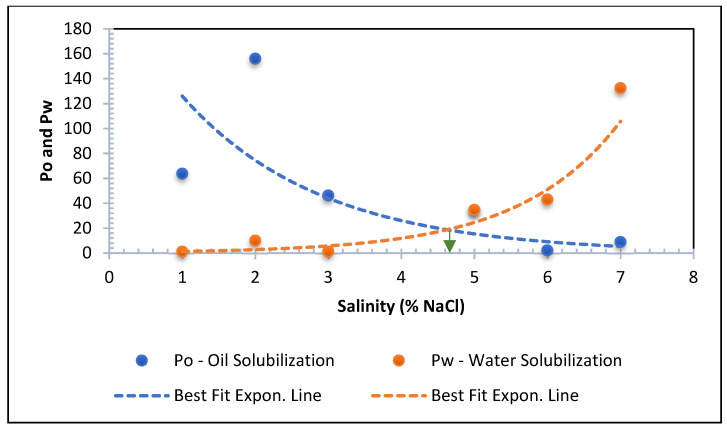
Optimum salinity determination.

Figure 8 indicates the phase volumes at a fixed surfactant concentration with an increasing NaCl concentration. The graph shows that the amount of micro-emulsion fluctuated with increasing NaCl. The figure demonstrates that the emulsion volume increases at the expense of oil phase and moves to the upper phase region. The maximum micro-emulsion is reached at 1% and 3% salinities. It can be concluded that optimum salinity can be found from a 2% NaCl concentration. The solubilization parameters for both oil and brine in the sample was calculated using the Healy and Reed correlation according to equations from 1 to 4. Figure 9 demonstrates the optimum salinity determination technique. After eliminating out of range data, the solubilization parameters are plotted against salinity, and best fit lines are drawn to obtain the optimum salinity. The optimum salinity for a pure APG-Crude oil system determined from Figure 9 is at 4.6% NaCl. Note that the optimum salinity will change if the formulation varies, for example, by adding alcohol, ketone, or polymer. IFT determination is not presented here because the IFT values of the surfactant and co-surfactant formulation are calculated using a spinning drop instrument.

### 5.2. IFT Measurement

#### IFT Values of Formic Acid with APG

**Figure 10:**
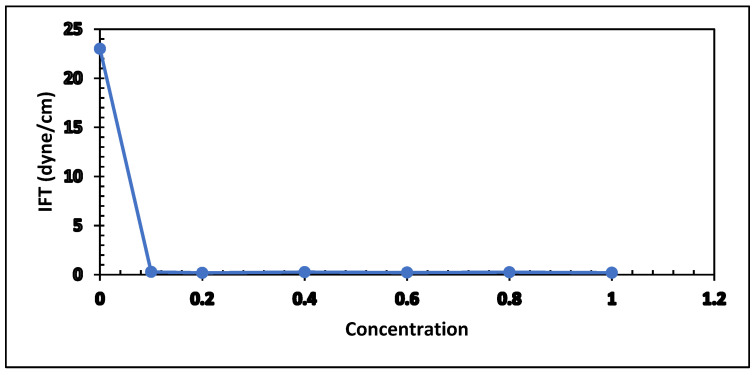
IFT measurements as a function of formic acid concentration.

The 0.50% of APG 264 with concentrations of formic acid ranging from 0.00% to 1.00% are measured and illustrated in Figure 10 and Table 4. The IFT declined sharply, approximately 22 dyne/cm from 23.00 dyne/cm to 0.19 dyne/cm, and then increased to 0.27 at 0.4% Formic acid. After that, it decreases to 0.20 dyne/cm at 1% formic acid concentration. The optimum concentration of formic acid was determined to be 0.60%.

**Figure 11:**
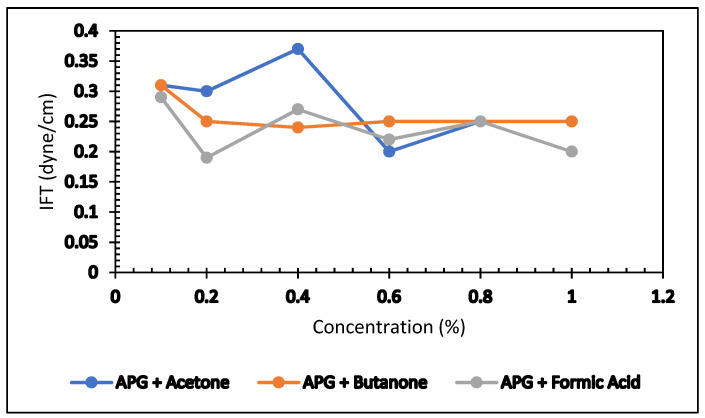
Comparison of IFT values.

IFT values of the acetone formulation (0.5% APG and 2% NaCl) and butanone solution (0.5% APG and 2 % NaCl) against Arabian light crude oil were measured using the same instrument, and the optimum point for acetone and butanone was selected. My previous paper titled “The Role of Microbial By-products in Green Enhanced Oil Recovery: Acetone and Butanone” [3] reported the detailed experimental process and result. In this article, a comparison of IFT value with formic was done and shown in Figure 11. Data are given in Table 5. It looks like the IFT behavior of formic acid, acetone, and butanone is similar.

### 5.3. Influence of Ketone Observed in Core Flood Experiment

#### 5.3.1. Acetone, APG 264, and Xanthan Gum Formulation

**Figure 12:**
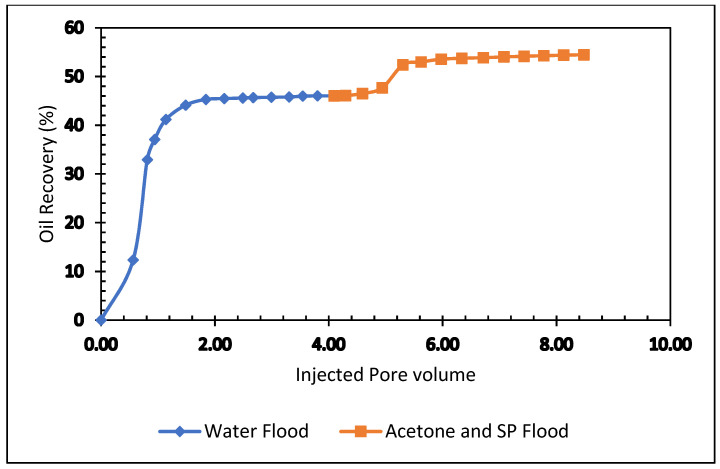
Total oil production from the carbonate core after the brine, acetone, and SP blend, followed by a post-flood with brine.

Two core flood experiments were performed to observe the oil recovery performance of acetone. Acetone of 0.50% was blended with 0.50% APG and 1000 mg/L (1000 ppm) XG. The results are shown in Table 6 and Figure 12.

In the beginning, the core flooding instrument was calibrated with known results. First, the sandstone core was flooded with brine and oil and kept to age seven days. Next, the test sample was run. Brine (2% NaCl) was flooded; the oil recovery was 46% of OIIP. In the tertiary stage, an acetone and SP blend was injected. The oil production was 8.44% after 2.4 pore volumes of injection. The total oil recovery from the sandstone was 54.44% at the end of the brine, APG slug, and post-flood with brine process. Figure 12 demonstrates the total oil recovery from the carbonate core.

#### 5.3.2. Butanone, APG 264, and Biopolymer Formulation

**Figure 13:**
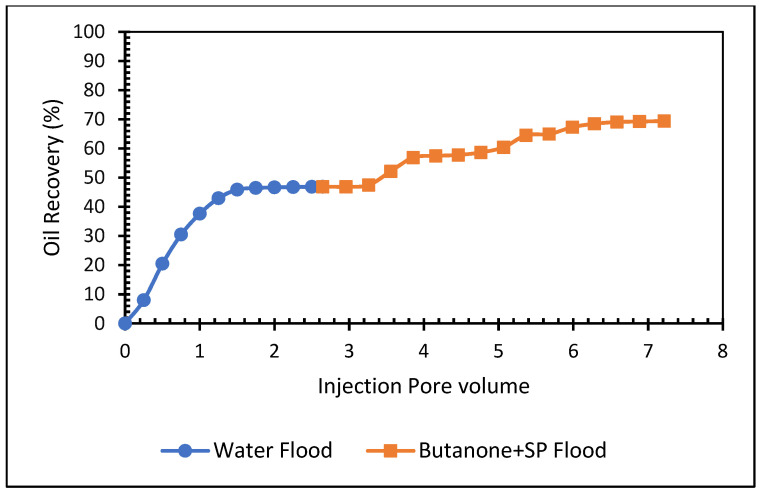
Total oil production from the carbonate core after brine, butanone, and SP mixture, followed by a post-flood with brine.

This experiment analyzed the effectiveness of butanone in the SP formulation in carbonate core. The formulation included 0.6% butanone, 0.5% APG 264, and 1000 mg/L XG. The core was vacuumed and saturated with brine (2% NaCl), then flooded with Arabian light crude oil and kept to age for seven days. First, the core was flooded with brine to recover residual oil saturation before the butanone-SP injection. The results of this flood are displayed in Figure 13 and Table 7. At the end of the waterflood, the oil recovery was 50%. Tertiary oil recovery from this formulation amounted to 23%. This was a significant amount of additional oil recovery. The total oil recovery of this formulation was 69% of OIIP. The APG, XG, and butanone combination are working effectively in the carbonate core.

### 5.4. Influence of Acrylic Acid in Carbonate

#### 5.4.1. Acrylic Acid, APG 264, and Xanthan Gum Formulation

**Figure 14:**
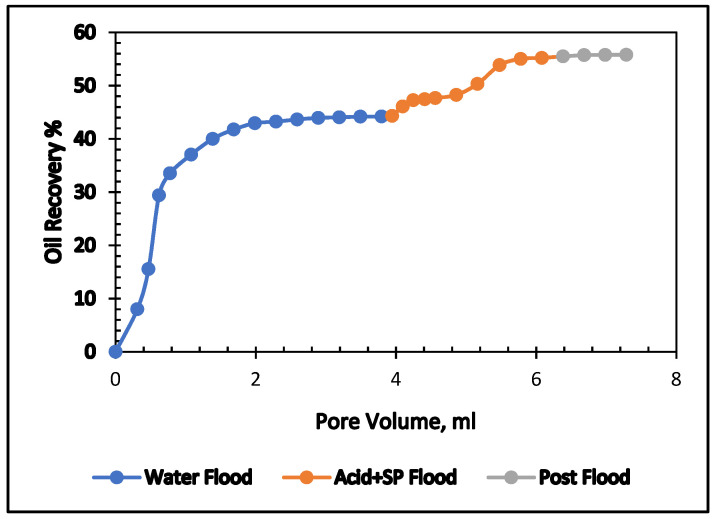
Total oil production from the carbonate core after brine, 0.4% Acrylic acid, and SP mixture, followed by a post-flood with brine.

**Figure 15:**
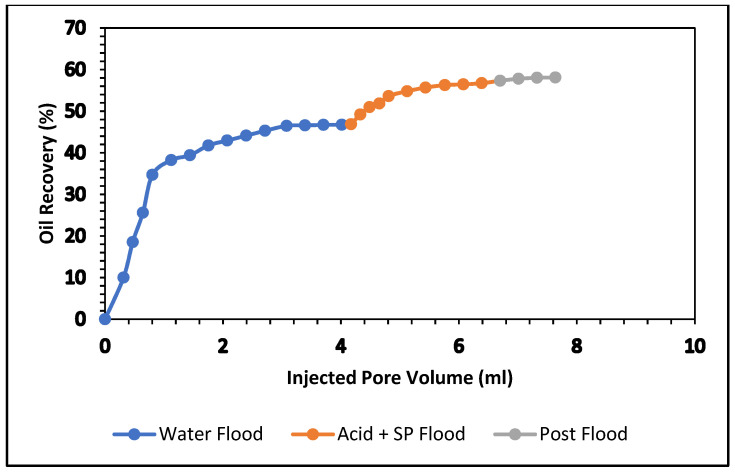
Total oil production from the carbonate core after brine, 0.5% Acrylic acid, and SP mixture, followed by a post-flood with brine.

The influence of acrylic acid in the surfactant and polymer mixture was examined in this experiment. We used two formulations: the first is 0.4% acrylic acid, and the second is 0.5% acrylic acid. Both were mixed with 0.5% APG and 1000 ppm (mg/L) and flooded in two carbonate cores. The results are shown in Table 8 and Figure 14 and Figure 15.

In the secondary stage, brine (2% NaCl) was flooded; the oil recovery was 44.20% of OIIP. In the tertiary stage, an acrylic acid and SP blend was injected. The oil production was 11.86% after 3.4 pore volumes of injection. The total oil recovery from the sandstone was 58.10% at the end of the brine, acrylic acid, SP slug, and post-flood with brine process. Figure 14 demonstrates the total oil recovery from the sandstone core. Figure 15 shows the oil recovery performance of 0.4% acrylic acid and SP formulation as a function of the pore volume injected. At the end of the waterflood, the oil recovery was 47%. Tertiary oil recovery from this formulation amounted to 58%. It seems that both formulations can provide the same amount of tertiary oil.

#### 5.4.2. Acrylic Acid, Butanol, APG 264, and Xanthan Gum Formulation

**Figure 16:**
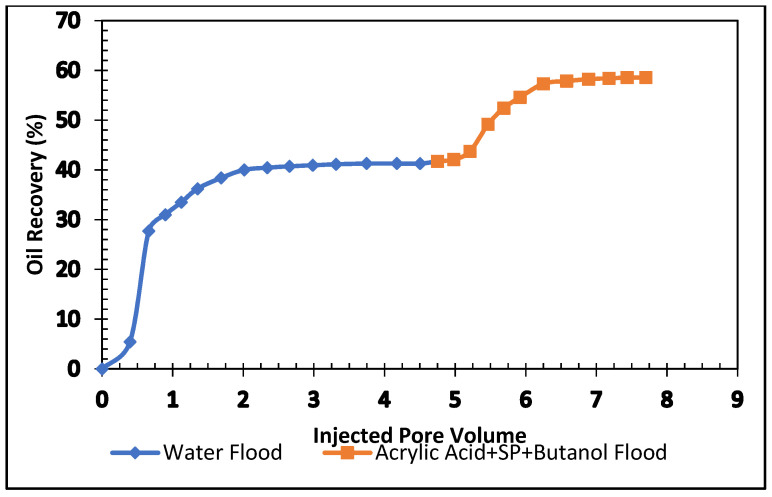
Total oil production from the carbonate core after brine, 0.5% Acrylic acid, butanol and SP mixture, followed by a post-flood with brine.

The experiment investigated the effect of butanol in acrylic acid and SP blend. In this formulation, 0.5% butanol was mixed with 0.5% acrylic acid, 0.5% APG and 1000 ppm (mg/L) and flooded in the carbonate core. The results are shown in Table 9 and Figure 16. In the secondary stage, the oil recovery was 41% of OIIP. In the tertiary stage, butanol, acrylic acid, and SP blend were injected, and the oil production was 18% after 3.0 pore volumes of injection. The total oil recovery from the sandstone was 59% at the end of the brine, butanol, acrylic acid, SP slug, and post-flood with brine process. Figure 16 demonstrates the total oil recovery from the sandstone core.

### 5.5. Comparison

#### Comparison of the Ketones, Acrylic Acids, Butanol, and SP Formulations

**Figure 17:**
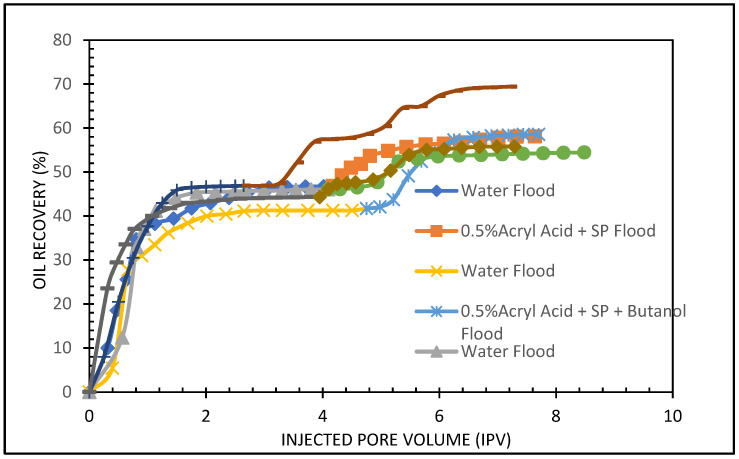
Total oil recovery observed from the five formulations.

**Figure 18:**
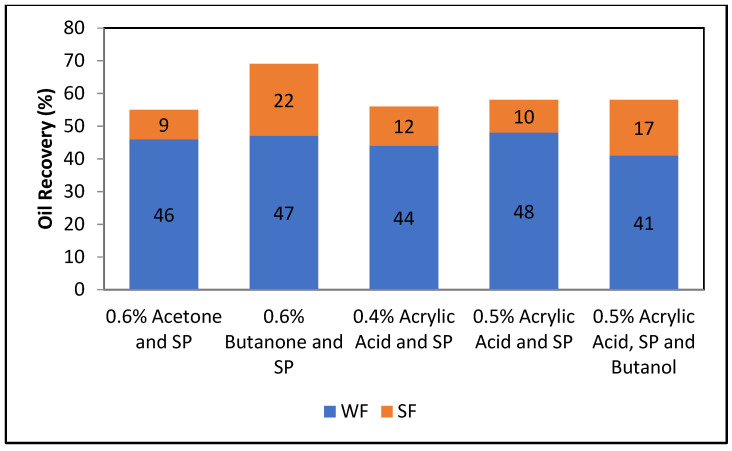
Comparison of total oil recovery observed from the five formulations.

Five core floods were conducted to examine the oil recovery performance in carbonate cores. The core flood results for the formulations applying carbonate cores are given in Table 10, and Figure 17 and Figure 18.

Figure 17 and Figure 18 compare total oil recovery from the five carbonate cores applying the five formulations listed in Table 10. It can be seen from Figure 18 that the tertiary oil recoveries are around 8% from the acetone and SP solution and 23% from the butanone and SP solution. Similarly, from the 0.4% and 0.5% acrylic acid and SP solutions, tertiary oil recoveries are about 12% and 11%, respectively. However, the 0.5% acrylic acid, butanol and SP formulation produces approximately 18% tertiary oil from the carbonate core. It is clear from Figure 18 that the butanone and SP blend made 22% tertiary oil from the carbonate core. This formulation produced 14% higher than the acetone and SP mixture and 5% higher than the acrylic acid, butanol and SP blend. By comparing the five formulations, it can be concluded that the butanone and SP formulation is more efficient at recovering tertiary oil from the carbonate core.

### 5.6. Influence of Formic and Acetic Acids in Sandstone

**Figure 19:**
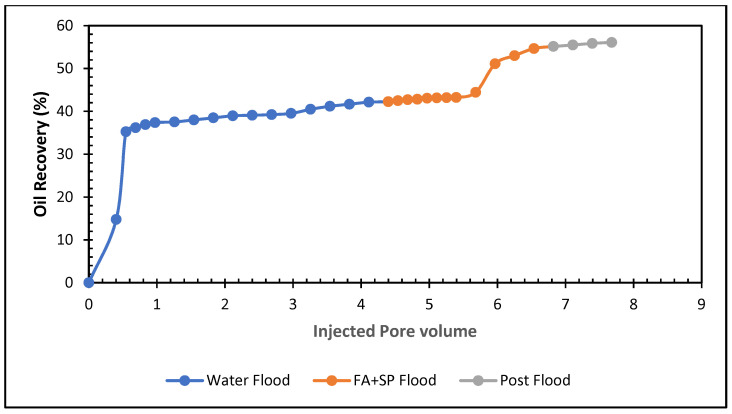
Total oil production from the carbonate core after brine, 0.5% formic acid, and SP mixture, followed by a post-flood with brine.

**Figure 20:**
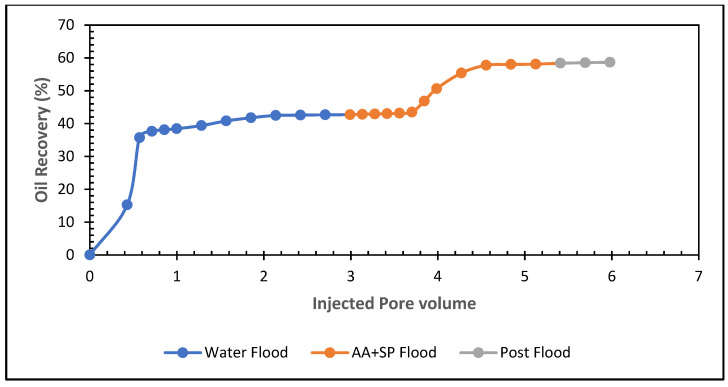
Total oil production from the carbonate core after brine, 0.5% acetic acid, and SP mixture, followed by a post-flood with brine.

The oil recovery potential of the green SP with formic and acetic acids formulations are also examined in the sandstone cores. The results of these combinations are listed in Table 11 and displayed in Figure 19 and Figure 20. Figure 19 demonstrates 0.5% formic acid, and SP produces ~13% tertiary oil from the sandstone core. The total oil recovery of this solution is ~55%.

However, the 0.5% acetic acid and SP mixture recovers about 15% residual oil and 59% total oil. This acetic acid and SP formulation are efficient in boosting tertiary oil from the sandstone core.

## 6. Conclusions

The work investigates the combined impact of green surfactant, polymer, organic acids, and ketones in boosting residual oil recovery from carbonate core via phase behavior modeling, IFT measurement, and core flood experiments. The main findings of this paper are highlighted below.

1. The sample’s solubilization parameters for oil and brine are calculated using the Healy and Reed correlation and plotted against salinity. The intersecting point of the two best-fit lines gives the optimum salinity. The optimum salinity for the APG–Crude oil system is determined at 4.6% NaCl.
2. A core flooding experiment in the carbonate core confirmed that a mixture of 0.5% APG, 1000 mg/L XG, and 0.6% acetone recovered ~8% residual oil. However, the same amount of APG and XG, blinding with 0.6% butanone, produced ~22% incremental oil, which was more than double crude oil. This formulation is efficient in recovering tertiary oil from carbonate core.
3. A combination of 0.5% APG, 1000 mg/L XG, and 0.5% acrylic acid gave ~11% incremental oil recovery. However, the same amount of APG, XG, and acrylic acid, mixing with 0.6% butanol, recovered ~18% tertiary oil. The SP, acrylic acid, and alcohol blend is effective in producing residual.
4. The blending of APG, XG with butanone is more effective in recovering residual crude than the mixture of APG, XG, acrylic acid, and butanol from the carbonate core.

## 7. Recommendations

1. It would be better to evaluate the oil recovery potential of formic acid and SP mixture and acetic acid and SP combination in carbonate core.
2. It recommended conducting wettability alteration analysis to understand this oil recovery controlling parameter [20,21].

## Figures and Tables

**Table 2:** Phase behavior study.

Salinity (%)	Initial Vol of Oil (mL)	Initial Vol of Slug (mL)	Final Oil Volume (mL)	Final Vol of Slug (mL)	Vol of Microemulsion (mL)	Phase Position
0.00	4.50	4.50	5.50	0	3.50	Upper
1.00	4.50	4.50	1.50	3.00	4.50	Upper
2.00	4.50	4.50	3.80	1.00	4.20	Upper
3.00	4.50	4.50	1.10	3.40	4.50	Upper
4.00	4.50	4.50	3.10	3.00	2.90	Middle
5.00	4.50	4.50	1.60	3.70	3.70	Middle
6.00	4.50	4.50	1.00	4.50	3.50	Lower
7.00	4.50	4.50	3.20	4.30	1.50	Lower

**Table 3:** Core properties.

Core Plug	Length (cm)	Diameter (cm)	Pore Volume (CC)	Dry Weight (gm)	Porosity (%)	Permeability (mD) Ka
C1	15.09	3.79	32	398.80	18.91	102.00
C2	15.5	3.80	33.24	400.16	19.94	91.16
C3	15.24	3.81	33.2	368.60	19.10	121.58
C4	15.24	3.81	32	372.38	18.40	185.97
C5	15.02	3.78	30.32	-	17.80	190.00
S6	15.05	3.79	35.04	355.45	20.17	124.2
S7	15.18	3.80	35.14	358.84	20.22	129.02

**Table 4:** APG surfactant and different concentration of formic acid.

Sample No.	Concentration (%)	IFT (dyne/cm)
1	2% NaCl	23.00
2	0.5% APG + 2% NaCl	0.285
3	0.5% APG + 0.2% Formic Acid + 2% NaCl	0.190
4	0.5% APG + 0.4% Formic Acid + 2% NaCl	0.266
5	0.5% APG + 0.6% Formic Acid + 2% NaCl	0.220
6	0.5% APG + 0.8% Formic Acid + 2% NaCl	0.250
7	0.5% APG + 1.0% Formic Acid + 2% NaCl	0.200

**Table 5:** IFT values of formic acid, acetone, and butanone with 0.5% APG and 2% NaCl.

Sample No.	Concentration of Acetone/Butanone/Formic Acid (%)	IFT (dyne/cm)		
		Acetone	Butanone	Formic Acid
1	0	23	23	23
2	0.1	0.31	0.31	0.29
3	0.2	0.3	0.25	0.19
4	0.4	0.37	0.24	0.27
5	0.6	0.2	0.25	0.22
6	0.8	0.25	0.25	0.25
7	1	0.25	0.25	0.2

**Table 6:** Flood results for acetone, APG 264, and Xanthan gum blend.

Formulation 1: 0.5% APG + 0.6% Acetone + 1000 mg/L XG + 2% NaCl Water								
Core	PV (cc)	Oil Volume (cc)	Soi (%)	Water Flood Recovery		S.P. Flood Recovery		Total
				cc	%	cc	%	%
C1	32	17	52.6	7.8	46	1.43	8.44	54.44

**Table 7:** Flood results for butanone, APG 264, and Xanthan gum mixture.

Formulation 2: 0.5% APG + 0.6% Butanone + 1000 mg/L XG + 2% NaCl Water								
Core	PV (cc)	Oil Volume (cc)	Soi (%)	Water Flood Recovery		S.P. Flood Recovery		Total
				cc	%	cc	%	%
C2	33.24	17	51.14	7.96	46.82	7.21	22.59	69.41

**Table 8:** Flood results for acrylic acid, APG 264, and Xanthan gum blend.

Formulation 3: 0.4% Acrylic Acid + 0.5% APG + 1000 mg/L XG + 2% NaCl Water Formulation 4: 0.5% Acrylic Acid + 0.5% APG + 1000 mg/L XG + 2% NaCl Water								
Core	PV (cc)	Oil Volume (cc)	Soi (%)	Water Flood Recovery		S.P. Flood Recovery		Total
				cc	%	cc	%	%
C3	33.24	17	51.2	7.514	44.20	2.02	11.86	56.06
C4	33	17	53.1	7.95	46.75	1.93	11.35	58.10

**Table 9:** Flood results for acrylic acid, butanol, APG 264, and Xanthan gum blend.

Formulation 5: 0.5% Acrylic Acid + 0.5% Butanol + 0.5% APG + 1000 mg/L XG + 2% NaCl								
Core	PV (cc)	Oil Volume (cc)	Soi (%)	Water Flood Recovery		S.P. Flood Recovery		Total
				cc	%	cc	%	%
C5	30.4	18.4	61	7.54	41	3.22	18	59

**Table 10:** Summary flood results of the five formulations.

Core	Formulation	Water Flood Recovery (%)	S.P. Flood Recovery (%)	Total (%)
C1	0.6% Acetone + 0.5% APG + 1000 mg/L XG + 2% NaCl	46	8.44	54.44
C2	0.6% Butanone + 0.5% APG + 1000 mg/L XG + 2% NaCl	46.82	22.59	69.41
C3	0.4% Acrylic Acid + 0.5% APG + 1000 mg/L XG + 2% NaCl	44.20	11.86	56.06
C4	0.5% Acrylic Acid + 0.5% APG + 1000 mg/L XG + 2% NaCl	46.75	11.35	58.10%
C5	0.5% Acrylic Acid + 0.5% Butanol + 0.5% APG + 1000 mg/L XG + 2% NaCl	41	18	59

**Table 11:** Flood results for acrylic acid, APG 264, and Xanthan gum blend.

Formulation 6: 0.5% Formic Acid + 0.5% APG + 1000 mg/L XG + 2% NaCl Water Formulation 7: 0.5% Acetic Acid + 0.5% APG + 1000 mg/L XG + 2% NaCl Water								
Core	PV (cc)	Oil Volume (cc)	Soi (%)	Water Flood Recovery		S.P. Flood Recovery		Total
				cc	%	cc	%	%
S6	35.04		59.93		42.2		12.9	55
S7	35.14		59.75		42.7		15.7	58.7

**Figure A2:**
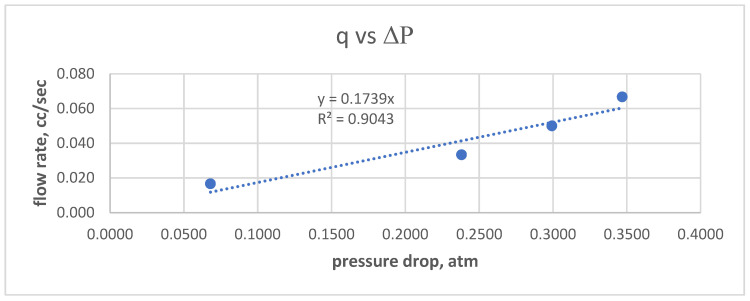
Average permeability calculation.

## Data Availability

Not applicable.

## Appendix A. Surfactant and Polymer Field Application

### Appendix A.1. Surfactant Flooding in Carbonates

**Table A1:** Field Application of Surfactant.

S/L	Surfactant	Synthetic/Bio/Green	Formation	Field	Country	Ref.
1	Petrostep-B100	Synthetic	carbonate	Cretaceceous upper Edwards	USA	[60]
2	Polyoxyethylene alcohol	Synthetic	carbonate	The cottonwood creek		[61,62]
3	A combination of petroleum sulfonate and alkylaryl ether sulphate	Synthetic		Bob slaughter block		[63]
4	Non-ionic ethoxy alcohol	Green	carbonate	Yates field		[61]
5	ORS-4l	Synthetic	sandstone	Tanner		[64]
6	Petroleum sulfonate, lignosulfonate, alkyl benzene sulfonate (ABS), petroleum carboxylate, bio-surfactant	Synthetic, Green and Bio	sandstone	Daging	China	[65,66,67,68,69]
7	Petroleum sulfonate	Synthetic	sandstone	Karamay		[70,71]
8	Petroleum sulfonate	Synthetic	sandstone	Viraj	India	[72]
9	Petrostep B-100		sandstone	West kiehl		[73]
10	Petroleum sulonate	Synthetic	sandstone	Minas	Indonesia	[74]
11			carbonates	Baturaja		[75]
12	SS-6066	Synthetic		Chihuido de la sierra negra	Argentina	[76]
13	Olefin sulfonate	Synthetic	sandstone	bramberge	Germany	[77]

### Appendix A.2. Polymer Flooding in Carbonates

**Table A2:** Field Application of Polymer.

S/L	Polymer	Synthetic/Bio	Formation	Field	Country	Ref
1	HPAM	Synthetic	Sandstone	North Burbank	USA	
2	HPAM	Synthetic	Sandstone	West Kiehli		[66]
3	PAM	Synthetic	Sandstone	Cambridge Minnelusa		[78]
4	PAM	Synthetic	Sandstone	Tanner		[65]
5	HPAM	Synthetic	Sandstone	Tambaredjo		[79]
6	HPAM	Synthetic	Sandstone	Daqing	China	[61]
7	HPAM	Synthetic	Sandstone	Gudong		[60]
8	HPAM	Synthetic	Sandstone	Xing Long Tai		[63]
9	Xanthan	Bio	Sandstone	Voador Offshore Feild	Brazil	[80]
10	HPAM	Synthetic	Sandstone	Viraj	India	[69]
11	PAM	Synthetic	Sandstone	Sanand		[70]
12	HPAM	Synthetic	Sandstone	Matzen	Austria	[73]
13	HPAM	Synthetic	Sandstone	Pelican Lake	Canada	[79]
14	PAM	Synthetic	Sandstone	David Pool		[75]
15	Xanthan	Bio	Sandstone	Eddesse-Nord	Germany	[80]
16	Hydroxyethylcellulose (HEC)	Bio	Sandstone	Russia Romashkino Field	Russia	[76,80]

### Appendix A.3. SP Flooding in Carbonates

**Table A3:** Application of the SP or ASP formulation in carbonate fields.

SP or ASP	Synthetic/Bio/Green	Field and Country	Formation	Oil Recovery %	Ref.
S: Petroleum sulfonate and alkyl ether sulfate P: PAM	Synthetic	Wichita County Regular Gunsight reservoir, Texas, USA	Carbonate	22.0	[81]
S: Petroleum sulfonates and Alkyl ether sulfate P: PAM	Synthetic	Wesgum field Smackover reservoir, Arkansas, USA	Carbonate	26.7	[82]
S: Alkyl ether sulfates and Witco petroleum sulfonate	Synthetic	Bob slaughter block lease San andres reservoir, Wyming, USA	Carbonate	12.0	[63]
Na2CO3 and anionic polymers	Synthetic	Isenhour, Wyming, USA	Carbonate	26.4	[83]
A: Na_2_CO_3_ S: Petrostep B-100 P: Alcoflood1175A	Synthetic	Cambridge Minnelusa, Wyming, USA	Carbonate	36.0	[78]
S: nonionic ethoxy alcohol (Shell 91-8) and Stepan CS-460 anionic ethoxy sulfate surfactant	Synthetic	Yates field, san andres reservoir, Texas, USA	Carbonate	15.0	[84,85]
S: Nonionic polyoxyethylene alcohol (POA)	Synthetic	Cottonwood Creek field, Wyming, USA Bighorn basin	Carbonate	10.4	[31,32]
A: Sodium carbonate alkaline solution S: 0.5% of surfactants in 200 gallons per ft of diesel oil and 0.5% of surfactants in 55 gallons per ft of xylene.	Synthetic	Mauddud carbonate reservoir, Bahrain	Carbonate	10–15	[86]
S: Nonionic surfactants	Synthetic	Semoga field, baturaja Formation, Indonesia	Carbonate	58,000 bbl per month	[70]

**Table A4:** Laboratory studies of the SP and ASP method in carbonate.

SP or ASP Formulation	Synthetic/Bio/Green	Reservoir	Permeability (mD)	Oil Recovery %	Ref.
Amphoteric petrostep B-100 surfactant and pusher 700E Polymer and sodium tripolyphosphate and sodium carbonate alkali.	Synthetic	Cretaceous Upper Edwards Reservoir Carbonate Formations From central Texas	75	45	[60]
Cationic surfactants of the type tetra alkyl ammonium Anionic surfactants	Synthetic	Outcrop chalk From stevns Klint Copenhagen	2–7	10–75	[87]
Anionic (ethoxylated and propoxylated sulfate) surfactants and sodium carbonate alkali mixture.	Synthetic	Dolomite cores	40–122	40–50	[21]
Nonionic ethoxy alcohol surfactants	Synthetic	Dolomite cores		-	[88]
Anionic ethoxylated and propoxylated sulfate surfactants Cationic (CTAB) surfactants	Synthetic	Calcite, Lithographic Limestone, Marble Dolomite plates		35–55	[89]
Cationic C12TAB surfactants	Synthetic	Outcrop chalk from Stvens Klint Copenhagen	1–3	50–90	[90]
Cationic C12TAB surfactants	Synthetic	Outcrop chalk	2–3	20–60	[91]
Five anionic (sulfonate, disulfonate and sulfate) surfactants	Synthetic	Calcites plates limestones cores	15	60–75	[92]
Anionic (sulfonate, disulfonate and sulfate) surfactants	Synthetic	Calcites plates limestones cores	15	30–50	[93]
Two anionic surfactants (ethoxylated sulfonate: AV-70, AV-150) Three nonionic surfactants (NP ethoxylate, 15-s-ethoxylate, TDA 30EO) Four cationic surfactants (CTAB, DTAB, Arquad C-50, Arquad T-50) surfactants	Synthetic	Limestone		70–80	[94]
Anionic surfactants: alkyl propoxy sulfates and their blends with internal olefin sulfonates, alkyl xylene sulfonates	Synthetic	Silurian dolomite outcrop cores	195	26–80	[95]
Two anionic and two nonionic surfactants	Synthetic	Siliceous and carbonate shale cores		-	[96]
Anionic Guerbet alkoxy carboxylate (GAC) surfactants	Synthetic	Silurian dolomite (478 mD) Estaillade limestone core	478 and 187	90–94.5	[97]
Nonionic branched nonylphenol ethoxylates (Huntsman SURFONICS N-120 & Huntsman SURFONICS N-150) and branched isotridecyl ethoxylate (Huntsman SURFONICS TDA-9) surfactants	Synthetic	SACROC carbonate cores	13–16	-	[98]

## Appendix B. Core Properties Measurement

### Appendix B.1. Properties and Calculation Procedure of Core Sample 4

The Indiana limestone core of 6-inch length and 1.5-inch diameter was used in this experiment. First, the core plug was cut, dried, and vacuumed, and then properties were measured. Calculation only sample 4 is described here.

### Appendix B.2. Pore Volume

Weight before saturation = 372.38 g

Weight after saturation = 404.6 g

$$\mathrm{Pore}\;\mathrm{Volume}=\frac{W_{\mathrm{after}}-W_{\mathrm{after}}}{\mathrm{density}\;}=\frac{404.6-372.38}{1.0125}=32{\;\mathrm{cm}}^{3}$$

### Appendix B.3. Porosity

$$\;V_{b}=\frac{\pi }{4}D^{2}L=\frac{\pi }{4}\times {\left(1.5\times 2.54\right)}^{2}\times \left(6\times 2.54\right)=173.75\;cm^{3}\;$$

$$\emptyset =\frac{V_{p}}{V_{b}}=\frac{32}{173.75}=18.4\%\;$$

### Appendix B.4. Permeability

**Table A5:** Permeability variations with different flow rates.

Flow Rate (cc/min)	Inlet Pressure (psi)	Outlet Pressure (psi)	Permeability (mD)
4	1017.4	1012.3	205.49
3	1040.4	1036	178.67
2	1051.3	1048.8	149.71
1	1099.7	1098.7	262.00

From Darcy’s law

$$K=\frac{1000*q*\mu *L}{A*∆P}$$

K: permeability, md

q: flow rate, cc/s

μ: viscosity, cp

L: length of the core, cm

A: area of the core, cm^2^

∆P: pressure drop, atm

Figure A2 shows that our core sample has an average permeability of 185.966 md.

### Appendix B.5. Saturation

**Table A6:** Saturation calculations.

PV (mL)	Brine Produced (mL)	Tube Volume (mL)	Brine Produced from Core (mL)	Soi	Swi
32	28	11	17	0.53	0.47

Volume of oil = 17 mL

$$\mathrm{oil}\;\mathrm{saturation}=\;\frac{V_{o}}{V_{p}}=\frac{17}{32}=0.53125$$

water saturation=1−oil saturation=1−0.53125=0.46875
